# Association of organ damage with predicted fat mass in a community-dwelling elderly: the Northern Shanghai study

**DOI:** 10.1007/s40520-023-02658-7

**Published:** 2024-02-12

**Authors:** Chong Xu, Shikai Yu, Song Zhao, Chen Chi, Ximin Fan, Jiadela Teliewubai, Jing Xiong, Jiamin Tang, Yifan Zhao, Yawei Xu, Yi Zhang

**Affiliations:** Department of Cardiology, Shanghai Tenth People’s Hospital, Tongji University School of Medicine, 301 Yanchang Road, Shanghai, 200072 China

**Keywords:** Fat mass, Organ damage, Elderly

## Abstract

**Background:**

Body fat mass (FM) is associated with multiple organ damage. However, data regarding the relationship between various organ damage and FM are rare in the elderly. Therefore, we aim to perform an analysis on the relationship between organ damage and FM in a geriatric cohort.

**Methods:**

3331 participants were included in this analysis. Based on age, body height, body weight, waist circumference, and race, we calculated FM with the established formula. Organ damage, including arterial stiffening, lower extremity atherosclerosis, left ventricular hypertrophy (LVH), micro-albuminuria, and chronic kidney disease (CKD), were measured and calculated with standard methods.

**Results:**

All organ damage parameters were significantly related to FM (all *p* < 0.001). In univariate logistics regression, the highest quartile of FM was tied to the increased risk of arterial stiffening, lower extremity atherosclerosis, LVH, micro-albuminuria, and CKD (all *p* < 0.05). After adjustment, participants with higher quantiles of FM had a significantly increased odd ratio (OR) for arterial stiffening [OR = 1.51, 95% confidence interval (CI): 1.15–1.99, *p* = 0.002] and LVH (OR = 1.99, 95% CI: 1.48–2.67, *p* < 0.001). Moreover, FM was linearly associated with arterial stiffening and LVH in total population and gender subgroups. Independent of confounders, FM was significantly correlated with arterial stiffening, lower extremity atherosclerosis, LVH and CKD in female, while was only related to LVH in male.

**Conclusions:**

Among various organ damage, elevated FM is significantly and independently associated with arterial stiffening and LVH in the elderly. Compared with men, women with increased FM are more likely to have multiple organ damage.

**Supplementary Information:**

The online version contains supplementary material available at 10.1007/s40520-023-02658-7.

## Introduction

With the rapid global economic and social development, people’s living condition have been dramatically improved. Along with this, the global prevalence of obesity has increased year by year in the past decades [1–3]. Obesity often leads to an increased risk of cardiovascular diseases such as hypertension, myocardial infarction, and stroke [4]. Therefore, it is necessary to accurately assess obesity. According to WHO guidelines, body mass index (BMI) is the most common indicator to define and diagnose obesity [1]. However, it does not accurately assess the real hazard of obesity, because both body fat mass (FM) and muscle mass can lead to increased BMI, but with differentiate clinical significance [5]. Furthermore, although dual-energy X-ray absorptiometry, computerized tomography and magnetic resonance imaging can accurately measure body fat, their clinical use is limited due to the high price and low accessibility [6]. Therefore, there is an urgent need to find easy, inexpensive and accurate metrics to assess body FM.

Previous studies have developed predictive formulas for whole-body FM from common anthropometric indicators, but factors such as limitation to a specific participant or lack of external validation have prevented these formulas from being widely used in clinical practice [7, 8]. Recently, Lee et al. developed a formula to predict whole-body FM using a large-scale, multi-ethnic population from the National Health and Nutrition Examination Surveys [9]. The gender-specific formula was calculated based on age, body height, body weight, waist circumference and race. Validation with internal cohorts, external cohorts and obesity-related biomarkers confirmed that the formula can be widely applied in epidemiological studies [10–12]. Besides, the accuracy and predictive power of this prediction equation has been demonstrated in Asian and Chinese populations [13–15]. Previous studies have found an association between whole-body FM and damage of blood vessels, heart, and kidneys [16–18]. However, there are few studies focusing on the analysis of the various organ damage and FM in elderly population. Therefore, we aimed to use the novel FM formulas to assess the relationship between various organ damage and FM in a community-dwelling elderly.

## Methods

### Participants

The Northern Shanghai Study is a prospective cohort study, focusing on the elderly in the northern community of Shanghai, China. The study has been registered on ClinicalTrials (NCT02368938). The detailed study protocol had been published [19]. The study recruited participants mainly through neighborhood committees, community hospitals and direct distribution of flyers. Inclusion criteria for the study were: (1) age equal to or greater than 65 years; (2) local residents in northern Shanghai; (3) willingness for long-term follow-up. Exclusion criteria were: (1) diagnosis of severe heart disease or end-stage renal disease; (2) life expectancy of less than 5 years or cancer; (3) stroke within 3 months; (4) breach of study protocol. “Life expectancy of less than 5 years” was defined as participants with serious diseases including cancer, heart disease, liver disease, kidney disease, and some chronic diseases such as diabetes, hypertension, and chronic respiratory disease combined with serious complications. From Jun 2014 to Sep 2019, 3590 residents were invited, of whom 3363 agreed to participate in the study. In addition, due to some missing data from 32 residents, we finally used data from 3331 participants for this analysis. Notably, the current analysis was a cross-sectional study using baseline data at the time of enrollment.

### Social, clinical and biological parameters

A standardized questionnaire was used to collect basic information about the residents, including gender, age, education, contact information, history of alcohol consumption, smoking, diabetes, renal insufficiency and cardiovascular disease. Body height, body weight, waist circumference (WC) and hip circumference were measured by the trained professionals. BMI was obtained by dividing weight (kg) by the square of height (m^2^). After abstaining from smoking and alcohol for at least 30 min prior to the measurement, blood pressure was measured using an Omron sphygmomanometer. We took three sitting blood pressure measurements and averaged them for next analysis. Hypertension was defined as systolic/diastolic blood pressure ≥ 140/90 mmHg or a history of antihypertensive medication. After the participants fasted overnight, venous blood and urine were collected. Then a series of tests were performed by the laboratory of Shanghai Tenth People’s Hospital, which included blood routine, kidney function, blood lipids, blood glucose and urine albumin–creatinine ratio (UACR). Diabetes was defined as fasting blood glucose ≥ 7.0 mmol/L or the presence of diabetes treatment.

### Predicted fat mass equations

The researchers used dual-energy X-ray absorptiometry as the gold standard to derive the predicted equations based on height, weight, and waist circumference [9]. The formula is as follows:

Women: Fat mass (kg) = 11.817 + 0.041*age (years)− 0.199*height (cm) + 0.610*weight (kg) + 0.044*WC (cm) + 0.388*Mexican + 0.073*Hispanic− 1.187*Black + 0.325*Other.

Men: Fat mass (kg) = − 18.592− 0.009*age (years)− 0.080*height (cm) + 0.226*weight (kg) + 0.387*WC (cm) + 0.08*Mexican− 0.188*Hispanic− 0.483*Black + 1.05*Other.

The Asian population in the current study belongs to other races, so the assignment values of the race variable for the current study population were 0.325 and 1.050 in the prediction equations for women and men, respectively.

### Measurements and definitions of organ damages

According to guideline recommendations, the carotid-femoral pulse wave velocity (cf-PWV) is the gold standard for assessing arterial stiffening. We used applanation tonometry (SphygmoCor, AtCor Medical, Australia) to measure it. Cf-PWV > 10 m/s was defined as arterial stiffening [20]. Peripheral arterial disease can be assessed by ABI, which is measured automatically with the VP-1000 device (Omron, Japan). ABI < 0.9 is diagnostic of lower extremity atherosclerosis [21]. Cardiac ultrasonography was performed by experienced cardiologists using the MyLab 30 Gold cardiovascular machine (ESAOTE SpA, Genoa, Italy). The left ventricular mass index (LVMI) was calculated by taking the left ventricular internal diameter (LVIDd), the septum (SWTd) and the end-diastolic posterior wall thickness (PWTd) with the following formula: LVMI (g/m^2^) = [0.8 × [1.04 × [(LVIDd + PWTd + SWTd)^3^− (LVIDd)^3^] + 0.6]/Body surface area. Left ventricular hypertrophy (LVH) was defined as LVMI ≥ 115 g/m^2^ in men and LVMI ≥ 95 g/m^2^ in women [22]. We calculated urine albumin to creatinine ratio (UACR) with the formula: UACR = urinary albumin/urinary creatinine. The UACR > 30 mg/g was diagnostic of micro-albuminuria. In addition, eGFR was calculated with the formula: eGFR = 175 × blood creatinine (mg/dl)^− 1.154^ × age (years)^− 0.203^ × 0.742 (women) [23]. eGFR ≤ 60 ml/min/1.73 m^2^ was defined as chronic kidney disease (CKD) [24].

### Statistical analysis

Continuous variables were expressed as mean ± standard deviation and categorical variables were expressed as absolute numbers (percentages). Differences between the different variables in men and women were compared by *t* test or chi-squared test. In addition, age-corrected spearman analysis was used to clarify the relationship between FM and organ damage parameters. Next, we divided the entire population into four groups based on FM tertile and put them into univariate and multivariate logistic regression models to analyze the relationship between FM and organ damage. The multivariate model included gender, age, hypertension, diabetes, high-density lipoprotein cholesterol (HDL-c), low-density lipoprotein cholesterol (LDL-c), smoking history and family history of early-onset cardiovascular disease. Finally, according to gender, subgroup analysis was performed to verify the relationship between FM and organ damage in a multivariate logistic regression model. Statistical differences were defined as *p* < 0.05. All statistical analyses were performed using SAS software (version 9.4, SAS Institute, Inc., Cary, North Carolina, USA).

## Results

### Baseline characteristics

Basic characteristics of the study group according to gender are presented in Table 1. The mean age of the overall population was 71.08 ± 6.0 years, 65.96% were diagnosed with hypertension and 22.46% were diagnosed with diabetes. The average BMI was 24.6 ± 3.6 kg/m2. In the analysis of BMI categorized according to the Chinese obesity guidelines [25], 42.36% of the participants were in the normal range (18.5–23.9 kg/m^2^), 39.81% were overweight (24.0–27.9 kg/m^2^), and 14.65% were obese (≥ 28 kg/m^2^). We also found that BMI was associated with FM (*r* = 0.806, *p* < 0.001).There was no difference in prevalence of hypertension and diabetes between men and women. Compared to female residents, male residents smoked more and had higher diastolic blood pressure and thicker WC. In terms of blood lipids, total cholesterol, triglycerides, LDL-c and HDL-c were higher in women. In antihypertension and hypoglycemic agents, no significant difference was noted between two groups. In terms of organ damage parameters, men had higher LVMI, and there were no gender differences in the remaining parameters. In addition, women had higher FM than men (*p* < 0.001).

**Table 1:** Characteristics of participants

	Overall (*n* = 3331)	Male (*n* = 1450)	Female (*n* = 1881)	*p* value
Age (years)	71.08 ± 6.0	71.10 ± 5.8	71.07 ± 6.1	0.87
Hypertension, *n* (%)	2197 (65.96)	965 (66.50)	1230 (65.50)	0.52
Diabetes mellitus, *n* (%)	748 (22.46)	333 (22.97)	415 (22.06)	0.54
Smoking habit, *n* (%)	822 (24.68)	786 (54.21)	36 (1.91)	< 0.001
Bachelor degree, *n* (%)	553 (16.60)	341 (23.52)	212 (11.27)	< 0.001
Family history of premature CVD, *n* (%)	716 (21.49)	274 (18.94)	442 (23.62)	0.001
SBP (mmHg)	135.5 ± 17.2	135.3 ± 16.3	135.6 ± 17.8	0.65
DBP (mmHg)	79.2 ± 9.6	80.3 ± 9.6	78.4 ± 9.6	< 0.001
Waist circumference (cm)	86.59 ± 9.85	88.39 ± 9.32	85.19 ± 10.02	< 0.001
Hip circumference (cm)	96.61 ± 7.19	96.88 ± 6.75	96.41 ± 7.51	0.06
BMI (kg/m^2^)	24.6 ± 3.6	24.5 ± 3.3	24.6 ± 3.8	0.64
Fasting glucose (mmol/L)	5.8 ± 1.8	5.8 ± 1.9	5.7 ± 1.7	0.17
Total cholesterol (mmol/L)	5.1 ± 1.0	4.8 ± 1.0	5.3 ± 1.0	< 0.001
Triglyceride (mmol/L)	1.6 ± 1.0	1.6 ± 1.0	1.7 ± 1.1	< 0.001
LDL-c (mmol/L)	3.1 ± 0.9	3.0 ± 0.9	3.3 ± 0.9	< 0.001
HDL-c (mmol/L)	1.40 ± 0.37	1.29 ± 0.34	1.48 ± 0.37	< 0.001
Treatments				
Antihypertension agents, *n* (%)	1662 (49.89)	729 (50.28)	933 (49.6)	0.70
Hypoglycemic agents, *n* (%)	532 (15.97)	242 (16.69)	290 (15.42)	0.32
Insulin therapy, *n* (%)	114 (3.42)	54 (3.72)	60 (3.19)	0.40
Statin therapy, *n* (%)	614 (18.43)	245 (16.90)	369 (19.62)	0.04
Organ damage				
Cf-PWV (m/s)	9.32 ± 2.29	9.29 ± 2.34	9.35 ± 2.26	0.47
ABI	1.03 ± 0.12	1.03 ± 0.13	1.03 ± 0.12	0.80
LVMI (g/m^2^)	87.7 ± 28.5	89.4 ± 28.7	86.4 ± 28.3	0.003
UACR (mg/g)	58.6 ± 144.5	54.4 ± 101.3	61.8 ± 170.6	0.12
eGFR (ml/min/1.73/m^2^)	80.12 ± 18.78	80.20 ± 20.03	80.06 ± 17.78	0.84
Fat mass (kg)	21.38 ± 6.37	18.10 ± 5.55	23.92 ± 5.77	< 0.001

*CVD* cardiovascular disease, *SBP* systolic blood pressure, *DBP* diastolic blood pressure, *BMI* body mass index, *LDL-c* low-density lipoprotein cholesterol, *HDL-c* high-density lipoprotein cholesterol, *Cf-PWV* carotid-to-femoral pulse wave velocity, *ABI* ankle-to-brachial index, *LVMI* left ventricular mass index, *UACR* urine albumin to creatinine ratio, *eGFR* estimated glomerular filtration rate

### Correlation analysis of organ damage parameters and FM

As shown in Table 2, after correcting for age, FM was related with cf-PWV (*r* = 0.185, *p* < 0.001), ABI (*r* = − 0.133, *p* < 0.001), LVMI (*r* = 0.120, *p* < 0.001), UACR (*r* = 0.098, *p* < 0.001) and eGFR (*r* = − 0.063, *p* < 0.001) in whole population.FM was correlated with cf-PWV (*r* = 0.180, *p* < 0.001), ABI (*r* = − 0.075, *p* = 0.006), LVMI (*r* = 0.205, *p* < 0.001), UACR (*r* = 0.081, *p* = 0.003) in men residents. In women, FM was related to cf-PWV (*r* = 0.202, *p* < 0.001), ABI (*r* = − 0.182, *p* < 0.001), LVMI (*r* = 0.156, *p* < 0.001), eGFR (*r* = − 0.099, *p* < 0.001).

**Table 2:** Age-adjusted correlation analysis between organ damage parameters and fat mass

	Overall		Male		Female	
	*r*	*p*	*r*	*p*	*r*	*p*
Cf-PWV, m/s	0.185	< 0.001	0.180	< 0.001	0.202	< 0.001
ABI	− 0.133	< 0.001	− 0.075	0.006	− 0.182	< 0.001
LVMI (g/m^2^)	0.120	< 0.001	0.205	< 0.001	0.156	< 0.001
UACR (mg/g)	0.098	< 0.001	0.081	0.003	0.043	0.073
eGFR (ml/min/1.73/m^2^)	− 0.063	< 0.001	− 0.042	0.126	− 0.099	< 0.001

*Cf-PWV* carotid-to-femoral pulse wave velocity, *ABI* ankle-to-brachial index, *LVMI* left ventricular mass index, *UACR* urine albumin to creatinine ratio, *eGFR* estimated glomerular filtration rate

### The relationship between the incidence of organ damage and FM

**Fig. 1:**
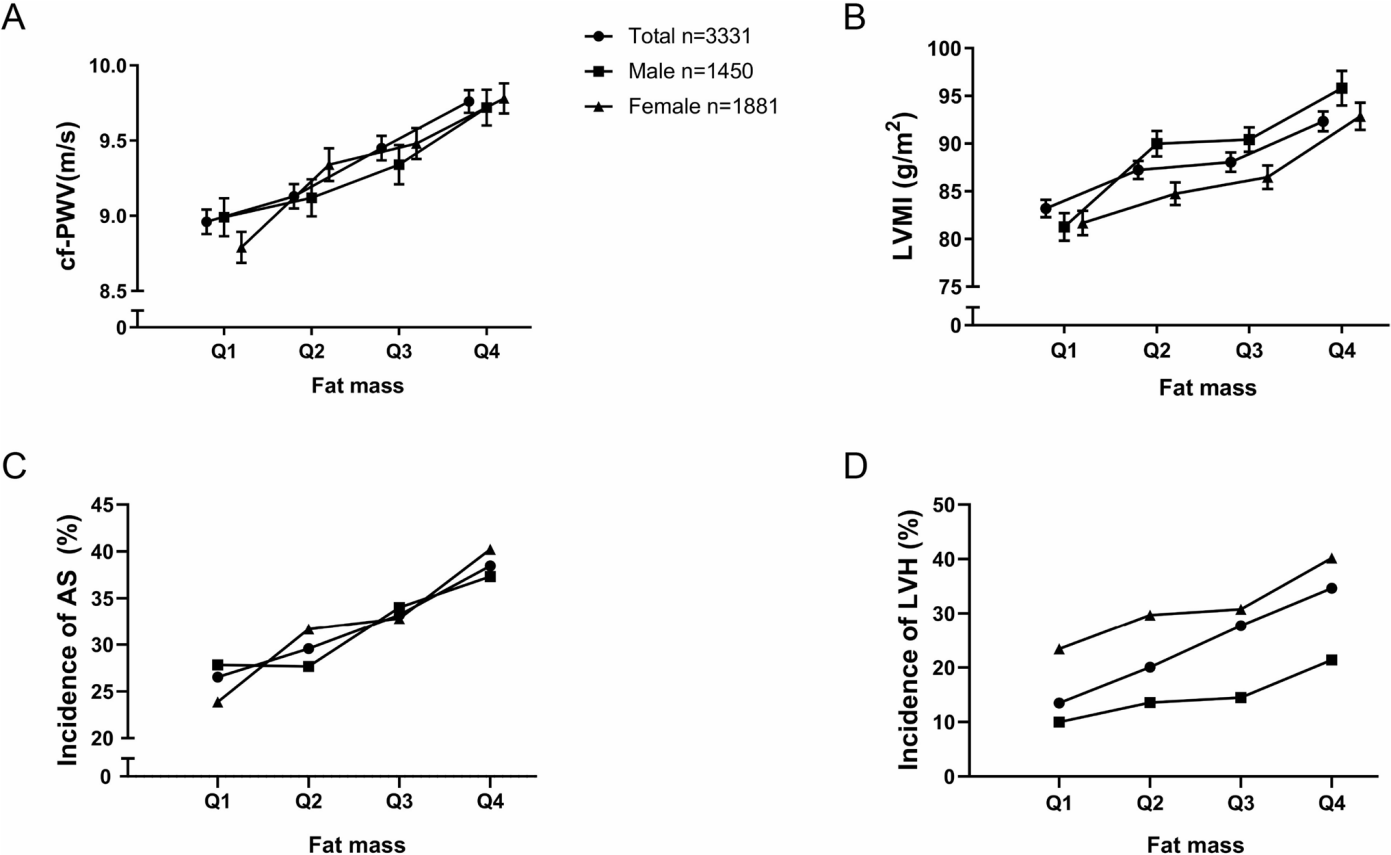
Trends of cf-PWV, LVMI and incidence of AS and LVH in quartiles with fat mass. In total or gender groups, residents in the upper quartile of fat mass have higher cf-PWV and LVMI (**A**, **B**). In total or gender groups, residents in the upper quartile of fat mass are more likely to have AS and LVH (**C**, **D**). *Cf-PWV* carotid-to-femoral pulse wave velocity, *LVMI* left ventricular mass index, *AS* arterial stiffening, *LVH* left ventricular hypertrophy, *FM* fat mass. Total: Q1: FM < 17.253, Q2: 17.253 ≤ FM < 21.0696, Q3: 21.0696 ≤ FM < 25.2245, Q4: FM ≥ 25.2245. Male: Q1: FM < 14.5918, Q2: 14.5918 ≤ FM < 17.9414, Q3: 17.9414 ≤ FM < 21.4165, Q4: FM ≥ 21.4165. Female: Q1: FM < 20.062, Q2: 20.062 ≤ FM < 23.4385, Q3: 23.4385 ≤ FM < 27.282, Q4: FM ≥ 27.282

Table 3 exhibits the relationship between overall FM and incidence of organ damage by univariate and multivariate logistics regression. In univariate analysis, compared to the lowest quartile, the highest quartile of FM was related to increased risks of arterial stiffening [OR = 1.73, 95% confidence interval (CI): 1.40–2.14, *p*_for trend_ < 0.001), lower extremity atherosclerosis (OR = 1.42, 95% CI: 1.07–1.87, *p*_for trend_ = 0.009), LVH (OR = 3.40, 95% CI: 2.66–4.35, *p*_for trend_ < 0.001), micro-albuminuria (OR = 1.42, 95% CI: 1.17–1.73, *p*_for trend_ < 0.001) and CKD (OR = 1.22, 95% CI: 0.90–1.67, *p*_for trend_ = 0.041). In the multivariate regression, residents in the highest quartile had an increased risk of arterial stiffening (OR = 1.51, 95% CI: 1.15–1.99, *p*_for trend_ = 0.002), LVH (OR = 1.99, 95% CI: 1.48–2.67, *p*_for trend_ < 0.001). Besides FM, age (OR = 1.05, 95% CI: 1.04–1.07), men (OR = 0.46, 95% CI: 0.35–0.59) and hypertension (OR = 1.68, 95% CI: 1.39–2.05) were also correlated with LVH (all *p* < 0.001). The relationship between multiple organ damage and all the parameters are shown in Table S1. Based on these results, we further analyzed the trends of cf-PWV, LVMI and the incidence of arterial stiffening, LVH in different quartiles of FM (Fig. 1). In total or gender groups, residents in the upper quartile of fat mass had higher cf-PWV (*p* < 0.05) and LVMI (*p* < 0.05). Similarly, participates in the upper quartile of fat mass were more likely to have arterial stiffening (*p* < 0.05) and LVH (*p* < 0.05).

**Table 3:** Association of organ damage in quartiles with fat mass in unadjusted and adjusted models

	Cf-PWV > 10 m/s	ABI < 0.9	LVH	MAU	CKD
Unadjusted					
Q1	1	1	1	1	1
Q2	1.16 (0.94–1.45)	0.72 (0.53- 0.99)	1.61 (1.24–2.09)	1.13 (0.93–1.37)	0.85 (0.61–1.19)
Q3	1.38 (1.11–1.71)	0.77 (0.57- 1.06)	2.46 (1.91–3.16)	1.28 (1.05–1.55)	1.31 (0.96–1.78)
Q4	1.73 (1.40–2.14)	1.42 (1.07- 1.87)	3.40 (2.66–4.35)	1.42 (1.17–1.73)	1.22 (0.90–1.67)
*p* for trend	< 0.001	0.009	< 0.001	< 0.001	0.041

Adjusted					
Q1	1	1	1	1	1
Q2	1.12(0.88–1.44)	0.71(0.51–1.00)	1.30(0.98–1.72)	1.03(0.83–1.27)	0.82(0.57–1.18)
Q3	1.28(0.99–1.67)	0.74(0.52–1.05)	1.69(1.28–2.25)	1.07(0.85–1.33)	1.16(0.81–1.67)
Q4	1.51(1.15–1.99)	1.35(0.94–1.93)	1.99(1.48–2.67)	1.06(0.83–1.35)	1.03(0.70–1.52)
*p* for trend	0.002	0.059	< 0.001	0.607	0.472

Q1: FM < 17.253, Q2: 17.253 ≤ FM < 21.0696, Q3: 21.0696 ≤ FM < 25.2245, Q4: FM ≥ 25.2245. Adjusted for age, gender, hypertension, diabetes, HDL-c, LDL-c, smoking habit, family history of premature CVD in the multivariable logistic regression

*Cf-PWV* carotid-to-femoral pulse wave velocity, *ABI* ankle-to-brachial index, *LVH* left ventricular hypertrophy, *MAU* micro-albuminuria, *CKD* chronic kidney disease, *FM* fat mass, *HDL-c* high-density lipoprotein cholesterol, *LDL-c* low-density lipoprotein cholesterol, *CVD* cardiovascular disease

**Fig. 2:**
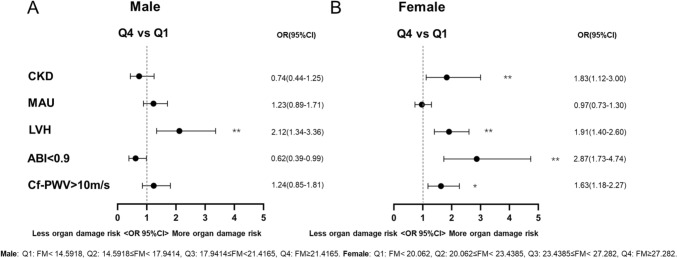
Association of organ damage in quartiles with fat mass in gender subgroups. In men, higher quartiles (Q4) of FM were only associated with an increased incidence of LVH (**A**). In women, participants with higher quartiles of FM were more likely to have arterial stiffening, lower extremity atherosclerosis, LVH and CKD, but not associated with MAU (B). OR odds ratios, CI confidence interval, Cf-PWV carotid-to-femoral pulse wave velocity, ABI ankle-to-brachial index, LVH left ventricular hypertrophy, MAU micro-albuminuria, CKD chronic kidney disease, FM fat mass, HDL-c high-density lipoprotein cholesterol, LDL-c low-density lipoprotein cholesterol, CVD cardiovascular disease. Male: Q1: FM < 14.5918, Q2: 14.5918 ≤ FM < 17.9414, Q3: 17.9414 ≤ FM < 21.4165, Q4: FM ≥ 21.4165. Female: Q1: FM < 20.062, Q2: 20.062 ≤ FM < 23.4385, Q3: 23.4385 ≤ FM < 27.282, Q4: FM ≥ 27.282. Adjusted for age, hypertension, diabetes, HDL-c, LDL-c, smoking habit, family history of premature CVD in the multivariable logistic regression. **: *p* < 0.01 *: 0.01 ≤ *p* < 0.05 statistical significance between the upper quartile (Q4) and the lowest quartile (Q1)

Next, we performed a subgroup analysis by gender. As shown in Fig. 2 and Tables S2 and S3, in women, participants with higher quartiles of fat mass were more likely to have arterial stiffening (OR = 1.63, 95% CI: 1.18–2.27, *p*_for trend_ = 0.011), lower extremity atherosclerosis (OR = 2.87, 95% CI: 1.73–4.74, *p*_for trend_ < 0.001), LVH (OR = 1.91, 95% CI: 1.40–2.60, *p*_for trend_ < 0.001) and CKD (OR = 1.83, 95% CI: 1.12–3.00, *p*_for trend_ = 0.008), but not associated with micro-albuminuria. In men, higher quartiles of fat mass were only associated with an increased incidence of LVH (OR = 2.12, 95% CI: 1.34–3.36, *p*_for trend_ < 0.001).

## Discussion

Based on the geriatric cohort from the northern Shanghai community, there were two important findings. First of all, after adjustment for cardiometabolic factors, higher levels of FM were still associated with higher risk of arterial stiffening and LVH among the elderly. On the other hand, in the elderly women, elevated FM was associated with more organ damage, including arterial stiffening, lower extremity atherosclerosis, LVH and CKD, while was only related to LVH in men.

Several studies have shown that older adults were prone to an increase in body fat [26, 27]. Excessive accumulation of fat can lead to structural and functional changes in vasculature [28]. Arterial stiffening is one of vascular damage and can predict cardiovascular events [29]. Nonetheless, the relationship between body fat and arterial stiffening was inconsistent. In a longitudinal cohort of Whitehall II study, findings showed that higher fat mass percent could predict arterial stiffening [30]. However, Nosrati et al. found no significant correlation between whole-body FM and cf-PWV in healthy adults [31]. In the current study, we found that FM and cf-PWV were correlated in the elderly. The difference about foregoing results may be due to the different population and health status. The possible mechanism is that excess fat can impair NO pathway in endothelial cells, which leads to vascular stiffness [32]. Besides, gender differences between FM and arterial stiffening were also observed in this study. Higher FM was associated with increased cf-PWV only in older women. Similarly, it is controversial whether obese patients are more susceptible to peripheral vascular disease [33, 34]. Our results found that older women who had higher FM were more likely to display lower extremity atherosclerosis. By contrast, the association was not observed in men. On the one hand, sex differences between FM and vascular damage may due to the higher frequency of smoking habit in men. Because smoking is an independent predictor of arteriosclerosis and atherosclerosis, it can increase the risk of vascular diseases, which may weak the effect of FM [35–37]. On the other hand, the withdrawal of estrogen may also make women participants more susceptible to vascular damage [38]. Unfortunately, our study did not test the levels of hormones. Further studies could clarify the relationship between FM, hormones and vascular damage.

There is growing evidence linking obesity to cardiac structural and functional abnormalities [39, 40]. Wong et al. defined obesity by BMI and found that obese patients without significant heart diseases were more likely to have early ventricular structure changes compared with nonobese patients [41]. Furthermore, in a biracial elderly cohort study, body fat measured by bioelectric impedance was associated with subclinical cardiac structural abnormalities [42]. Our results demonstrated a similarly strong association between higher FM and LVH in elderly men and women. Animal experiments also found that increased leptin could lead to cardiac inflammation and fibrosis in obese mice [43, 44]. In most cases, LVH is a compensatory mechanism for the heart to cope with left ventricular overload, which eventually leads to left ventricular dysfunction and heart failure [45]. These results can provide robust evidence that older people with increased FM are more prone to the early state of heart failure. Further follow-up should be carried out to assess the effect of FM on the incidence of heart failure.

Renal dysfunction is also one of the frequent organ impairments in the elderly [46]. The existing literature illuminated obesity could increase the risk of CKD [47]. A recent meta-analysis showed an association between elevated BMI and the risk of low eGFR in the general adult population [48]. Another study demonstrated metabolic syndrome was related to CKD in 616 non-diabetic older adults [49]. However, the relationship between body fat and renal function, other than BMI or WC, has not been widely elucidated in the elderly. We found that elevated FM was associated with an increased incidence of CKD in elderly women, but not men. This result is similar to the study by Yu et al. [18]. The potential mechanism for the difference may be large variability of muscle mass in men, which affects the accuracy of serum creatinine [50]. Further research can apply plasma cystatin C or others biomarkers to evaluate eGFR in men.

There are several major strengths of this study. First, the sample size of the North Shanghai study is sufficient to reduce the occurrence of random errors. Second, we used standardized questionnaires and measurements to ensure the accuracy of the risk factors. Finally, we analyzed the relationship between FM and multiple organ damage in the elderly, which had implications for body fat management in older adults.

Nonetheless, there are some limitations in this study. First, this study is a cross-sectional study and causality cannot be determined. However, the North Shanghai cohort will be followed up, which can provide some prospective data about the impact of FM on adverse cardiovascular events in an elderly population. Second, we used a predictive formula to calculate FM and did not apply gold standard tests such as DXA. However, Lee and colleagues have demonstrated a strong correlation between predicted FM and obesity-related markers. Third, the participants were Chinese, and the results may not be generalizable to other ethnic groups.

## Conclusion

Elevated FM is significantly and independently associated with arterial stiffening and LVH in the geriatric cohort. Compared with men, women with increased FM are more likely to have multiple organ damage.

### Supplementary Information

Below is the link to the electronic supplementary material.

Supplementary file1 (DOCX 30 KB)

## Data Availability

The datasets can be available from the corresponding author on reasonable request.
