# Supplementary material for: Association of organ damage with predicted fat mass in a community-dwelling elderly: the Northern Shanghai study
Source: Aging Clin Exp Res. 2024 Feb 12;36(1):35. doi: 10.1007/s40520-023-02658-7 (PMC10861618; doi:10.1007/s40520-023-02658-7)
Supplement: Supplementary file 1 — Supplementary file1 (DOCX 30 KB) [file 40520_2023_2658_MOESM1_ESM.docx]

**Table S1** Association of organ damage with all the parameters in adjusted models

| Variables | Cf-PWV>10m/s | ABI<0.9 | LVH | MAU | CKD |
| --- | --- | --- | --- | --- | --- |
| Age, y | 1.11(1.10-1.13)^a^ | 1.10(1.08-1.12)^a^ | 1.05(1.04-1.07)^a^ | 1.05(1.04-1.07)^a^ | 1.14(1.12-1.16)^a^ |
| Gender (men=1, women=0) | 1.14(0.90-1.44)^b^ | 1.08(0.78-1.49)^b^ | 0.46(0.35-0.59)^a^ | 0.64(0.52-0.79)^b^ | 0.90(0.65-1.26)^b^ |
| HDL-c, mmol/L | 0.83(0.65-1.06)^b^ | 0.40(0.28-0.58)^a^ | 0.92(0.71-1.18)^b^ | 1.17(0.95-1.44)^b^ | 0.38(0.26-0.56)^a^ |
| LDL-c, mmol/L | 1.10(1.01-1.22)^a^ | 1.24(1.10-1.39)^a^ | 0.92(0.84-1.01)^b^ | 0.96(0.89-1.04)^b^ | 1.10(0.96-1.24)^b^ |
| family history of premature CVD (yes=1, no=0) | 0.86(0.70-1.06)^b^ | 0.85(0.64-1.14)^b^ | 1.02(0.83-1.25)^b^ | 1.02(0.86-1.22)^b^ | 1.02(0.76-1.37)^b^ |
| Smoking (yes=1, no=0) | 0.95(0.75-1.21)^b^ | 1.54(1.13-2.10)^a^ | 1.02(0.77-1.35)^b^ | 1.30(1.05-1.60)^a^ | 1.23(0.88-1.71)^b^ |
| Hypertension (yes=1, no=0) | 2.46(2.03-2.97)^a^ | 1.10(0.85-1.42)^b^ | 1.68(1.39-2.05)^a^ | 1.56(1.34-1.83)^a^ | 1.66(1.25-2.20)^a^ |
| Diabetes (yes=1, no=0) | 2.35(1.95-2.83)^a^ | 1.51(1.18-1.93)^a^ | 1.20(0.98-1.46)^b^ | 1.74(1.46-2.07)^a^ | 1.04(0.80-1.37)^b^ |

**Notes:** Adjusted for age, gender, hypertension, diabetes, HDL-c, LDL-c, smoking habit, family history of premature CVD, FM in the multivariable logistic regression.

a (P<0.05) b (P>0.05)

**Abbreviations:** Cf-PWV, carotid-to-femoral pulse wave velocity; ABI, ankle-to-brachial index; LVH, left ventricular hypertrophy; MAU micro-albuminuria; CKD chronic kidney disease; FM: fat mass; HDL-c, high-density lipoprotein cholesterol; LDL-c, low-density lipoprotein cholesterol; CVD: cardiovascular disease.

**Table S2.** Association of organ damage with FM by logistic regression analysis in male

|  | Cf-PWV>10m/s | ABI<0.9 | LVH | MAU | CKD |
| --- | --- | --- | --- | --- | --- |
| **Unadjusted** |  |  |  |  |  |
| Q1 | 1 | 1 | 1 | 1 | 1 |
| Q2 | 0.99(0.71-1.38) | 0.75(0.48-1.16) | 1.42(0.90-2.24) | 1.20(0.89-1.61) | 0.96(0.59-1.55) |
| Q3 | 1.34(0.97-1.84) | 0.94(0.62-1.44) | 1.53(0.97-2.41) | 1.33(0.99-1.78) | 1.04(0.65-1.67) |
| Q4 | 1.54(1.12-2.13) | 0.95(0.63-1.45) | 2.46(1.60-3.76) | 1.48(1.10-1.98) | 1.21(0.77-1.92) |
| P for trend | 0.002 | 0.930 | <0.001 | 0.008 | 0.369 |
|  |  |  |  |  |  |
| **Adjusted** |  |  |  |  |  |
| Q1 | 1 | 1 | 1 | 1 | 1 |
| Q2 | 0.85(0.59-1.23) | 0.63(0.39-1.00) | 1.28(0.80-2.04) | 1.11(0.82-1.52) | 0.74(0.44-1.25) |
| Q3 | 1.15(0.80-1.65) | 0.74(0.47-1.16) | 1.35(0.84-2.16) | 1.20(0.88-1.65) | 0.75(0.45-1.26) |
| Q4 | 1.24(0.85-1.81) | 0.62(0.39-0.99) | 2.12(1.34-3.36) | 1.23(0.89-1.71) | 0.74(0.44-1.25) |
| P for trend | 0.113 | 0.100 | 0.001 | 0.188 | 0.331 |

**Notes:** Q1: FM< 14.5918, Q2: 14.5918≤FM< 17.9414, Q3: 17.9414≤FM<21.4165, Q4: FM≥21.4165. Adjusted for age, hypertension, diabetes, HDL-c, LDL-c, smoking habit, family history of premature CVD in the multivariable logistic regression.

**Abbreviations:** Cf-PWV, carotid-to-femoral pulse wave velocity; ABI, ankle-to-brachial index; LVH, left ventricular hypertrophy; MAU micro-albuminuria; CKD chronic kidney disease; FM: fat mass; HDL-c, high-density lipoprotein cholesterol; LDL-c, low-density lipoprotein cholesterol; CVD: cardiovascular disease.

**Table S3.** Association of organ damage with FM by logistic regression analysis in female

|  | Cf-PWV>10m/s | ABI<0.9 | LVH | MAU | CKD |
| --- | --- | --- | --- | --- | --- |
| **Unadjusted** |  |  |  |  |  |
| Q1 | 1 | 1 | 1 | 1 | 1 |
| Q2 | 1.48(1.11-1.98) | 1.45(0.87-2.42) | 1.38(1.03-1.85) | 0.97(0.75-1.26) | 1.24(0.77-2.01) |
| Q3 | 1.56(1.17-2.09) | 1.97(1.21-3.21) | 1.45(1.09-1.94) | 1.12(0.86-1.45) | 1.93(1.24-3.02) |
| Q4 | 2.15(1.61-2.86) | 3.36(2.13-5.31) | 2.19(1.65-2.91) | 1.10(0.85-1.42) | 2.01(1.29-3.14) |
| P for trend | <0.001 | <0.001 | <0.001 | 0.326 | <0.001 |
|  |  |  |  |  |  |
| **Adjusted** |  |  |  |  |  |
| Q1 | 1 | 1 | 1 | 1 | 1 |
| Q2 | 1.46(1.06-2.02) | 1.42(0.83-2.44) | 1.37(1.01-1.86) | 0.97(0.74-1.28) | 1.28(0.76-2.13) |
| Q3 | 1.35(0.97-1.87) | 1.59(0.94-2.70) | 1.33(0.98-1.83) | 1.09(0.82-1.44) | 1.83(1.12-2.99) |
| Q4 | 1.63(1.18-2.27) | 2.87(1.73-4.74) | 1.91(1.40-2.60) | 0.97(0.73-1.30) | 1.83(1.12-3.00) |
| P for trend | 0.011 | <0.001 | <0.001 | 0.970 | 0.008 |

**Notes:** Q1: FM< 20.062, Q2: 20.062≤FM< 23.4385, Q3: 23.4385≤FM< 27.282, Q4: FM≥27.282. Adjusted for age, hypertension, diabetes, HDL-c, LDL-c, smoking habit, family history of premature CVD in the multivariable logistic regression.

**Abbreviations:** Cf-PWV, carotid-to-femoral pulse wave velocity; ABI, ankle-to-brachial index; LVH, left ventricular hypertrophy; MAU micro-albuminuria; CKD chronic kidney disease; FM: fat mass; HDL-c, high-density lipoprotein cholesterol; LDL-c, low-density lipoprotein cholesterol; CVD: cardiovascular disease.
